# Comprehensive analysis of senescence-related genes and immune infiltration in intervertebral disc degeneration: a meta-data approach utilizing bulk and single-cell RNA sequencing data

**DOI:** 10.3389/fmolb.2023.1296782

**Published:** 2023-12-22

**Authors:** Ya-Jun Deng, Xin-Gang Wang, Zhi Li, Bo Wang, Jie Li, Jun Ma, Xiong Xue, Xin Tian, Quan-Cheng Liu, Jia-Yuan Liu, Ying Zhang, Bin Yuan

**Affiliations:** Department of Spine Surgery, Xi’an Daxing Hospital, Yanan University, Xi’an, China

**Keywords:** intervertebral disc degeneration, senescence-related genes, immune infiltration, single-cell, risk score

## Abstract

**Objectives:** This study aims to identify the key senescence genes and potential regulatory mechanisms that contribute to the etiology of intervertebral disc degeneration (IDD).

**Method:** We analyzed GSE34095 and GSE70362 datasets, identifying key senescence-related differentially expressed genes (DEGs) in IDD using lasso regression. Risk scores classified patients into high- and low-risk groups. We compared pathways, functions, and immune infiltration between these groups. Diagnostic ability was assessed using ROC curves and a nomogram predicted IDD incidence. In single-cell dataset GSE165722, we evaluated expression of key senescence-related DEGs.

**Results:** We identified 12 key senescence-related DEGs distinguishing high- and low-risk IDD patients. Enrichment analysis revealed cellular stress response, apoptotic signaling pathway, and protein kinase activation differences. Immune cell analysis showed elevated eosinophils in low-risk group and increased effector memory CD8 T, central memory CD4 T, myeloid-derived suppressor, natural killer, monocyte, Type 1 T helper, plasmacytoid dendritic, and natural killer T cells in high-risk group. A nomogram using AUC >0.75 genes (CXCL8, MAP4K4, MINK1, and TNIK) predicted IDD incidence with good diagnostic power. High senescence scores were observed in neutrophils.

**Conclusion:** Our diagnostic model, based on key senescence-related DEGs and immune cell infiltration, offers new insights into IDD pathogenesis and immunotherapy strategies.

## 1 Introduction

Lower back pain is one of the most common health problems, affecting up to 80% of people throughout their lives, causing severe disability worldwide, and resulting in significant medical costs (Qaseem et al., 2017). Intervertebral disc degeneration (IDD) is the leading cause of lower back pain (Cheng et al., 2018). Current treatments include medication, physical therapy, or surgical interventions that can partially relieve symptoms in the lower back and legs (Xin et al., 2022). They do not focus on replacing nucleus pulposus loss and restoring disc structure. This can lead to unsatisfactory results such as degeneration or recurrence of adjacent motion segments. Early diagnosis and timely treatment of IDD can effectively delay disease progression and significantly reduce the incidence of disability. Therefore, screening for diagnostic genes associated with IDD and elucidating their underlying pathogenesis could significantly prevent and treat IDD and may provide new avenues for the clinical treatment of IDD.

Senescence is a natural process, and there is a substantial link between senescence and IDD, which has been identified as a key factor in IDD (Cheng et al., 2022; Wu et al., 2022). Senescence leads to structural, metabolic, and biomechanical changes in the intervertebral disc that ultimately affect its function (Le Maitre et al., 2007). The senescent cells have several key features, such as sustained growth arrest, expression of anti-proliferative molecules (e.g., p16, INK4a) and activation of damage sensing signaling pathways (e.g., p38 MAPK and NF-kB) (He and Sharpless, 2017; Patil et al., 2019; Zhang et al., 2021). This in turn leads to the release of cytokines, chemokines, and other secreted phenotypic proteins associated with senescence, ultimately leading to inflammation and tissue degradation (Muñoz-Espín and Serrano, 2014). The senescence process is accompanied by changes in the immune microenvironment (Lanna et al., 2022). Studies have shown that immune dysregulation, including abnormal macrophage polarization, abnormal T-cell differentiation, abnormal expression of B cells, and improper T-cell differentiation are significantly associated with IDD (Song et al., 2022). Although both senescence and immune dysregulation are present in the IDD process, it is unclear if or how the two phenomena are linked. Therefore, identifying the molecular mechanisms of IDD senescence and further investigating the potential link between nucleus pulposus cell senescence and the immune microenvironment will provide new therapeutic concepts and alternative options for reversing the disease to provide better clinical treatment guidance.

Although previous study has provided preliminary evidence for the regulatory role of senescence-related genes in the development of IDD (Xu et al., 2023), they primarily focused on the biological functions of these genes in peripheral blood. The diagnostic value of these genes in nucleus pulposus tissue and their relationship with immune cells remain unclear. Thorough and comprehensive research on senescence-related genes in the nucleus pulposus tissue can enhance our understanding of IDD. Unlike previous publications that relied on experimental data generation, our research primarily focuses on meta-data analysis. We have extensively explored senescence-related biomarkers by publicly available datasets, including bulk and single-cell RNA sequencing data, through machine learning. In addition, we collected nucleus pulposus tissue samples for experimental verification of the analysis results to improve their accuracy and reliability. This approach allows us to assess a larger cohort and explore potential associations across various datasets, providing a broader perspective on IDD research.

## 2 Materials and methods

### 2.1 Bulk RNA data download

We downloaded two bulk RNA datasets (GSE34095 and GSE70362) from the GEO database (https:// www.ncbi.nlm.nih.gov/geo) via the GEOquery package (Davis and Meltzer, 2007). The GSE34095 dataset is from *Homo sapiens*, and the data platform is GPL96, which contains six samples, including three control and three disease samples (He et al., 2015). The GSE70362 dataset is from *H. sapiens*, and the data platform is GPL17810, containing 48 samples, including 16 control and 32 disease samples (Kazezian et al., 2015). The two datasets were standardized by limma package (Ritchie et al., 2015), and the entire samples of both datasets were de-batched using the sva package (Leek et al., 2012) after integrating the two datasets. The integrated data set consisted of 54 samples, 19 control and 35 IDD samples, and were included in this study.

### 2.2 Single cell data pre-processing and analysis

We downloaded single cell dataset GSE165722 from the GEO database, which contains eight samples, namely, S1 and S2 (Grade II), S3 and S4 (Grade III), S5 and S6 (Grade IV), and S7 and S8 (Grade V). Using the Seurat package (Hao et al., 2021), the expression matrix of the GSE165722 dataset was created as a Seurat object. We filtered the cells with >20% mitochondrial gene content as well as cells with features <200 or >4,000. We normalized the dataset’s sequencing depth using “SCTransform” to remove mitochondrial and cell cycle effects, identifying 3,000 highly variable genes. Principal Component Analysis (PCA) (David and Jacobs, 2014) was applied to identify significant principal components and visualize *p*-value distribution using the Elbowplot function. 15 principal components (PCs) were selected for t-SNE analysis to reduce dimensionality. We constructed K-nearest neighbors based on distances in PCA space using default parameters from the “FindNeighbors” function and 15 PC dimensions. Calling the “FindClusters” function divided cells into 17 clusters with a resolution of 0.5. Additionally, the “RunTSNE” function enabled dimensionality reduction for dataset visualization and exploration in cell annotation. Finally, AddModuleScore calculated scores for each cellular senescence.

### 2.3 Differential expression analysis

Differential analysis of genes in different groups was performed using the limma package. *p* < 0.05 was set as the threshold for DEGs. Where log_2_FC > 0 and log_2_FC < 0 mean they are upregulated or downregulated in the disease, respectively. The results of the difference analysis were presented by plotting heat maps with the pheatmap package (Kolde and Kolde, 2015) and volcano maps with ggplot2 (Wickham, 2009).

### 2.4 Senescence-related dataset acquisition

The senescence-related dataset REACTOME CELLULAR SENESCENCE v2023.1 was obtained from the MSigDB database (Liberzon et al., 2015) containing 198 genes and intersected with the DEGs obtained by differential analysis for subsequent analytical studies.

### 2.5 Model construction

Least absolute shrinkage and selection operator (LASSO) regression is a machine learning algorithm commonly used to build models today, using regularization to address the occurrence of overfitting during curve fitting and improve the accuracy of the model. We used the glmnet package (Simon et al., 2011) with integrated GSE34095 and GSE70362 for model construction with parameters set.seed 1) and family = “binomial” to further screen for variables associated with IDD based on key senescence-related DEGs. Finally, the risk score equation was calculated by optimizing the expression values of the genes and the associated regression coefficients:

$$\text{Riskscore}=\sum_{i=1}^{n}{coef}_{i}*\exp_{i}$$

The genes obtained by the LASSO algorithm were defined as key senescence-related DEGs.

### 2.6 Gene Ontology and Kyoto Encyclopedia of Genes and Genomes

The integrated GSE34095 and GSE70362 disease samples were divided into a high-risk score group and a low-risk score group. Differential analysis of genes in different groups was performed using limma. *p* < 0.05 was set as the threshold for DEGs. Gene Ontology (GO) analysis is a common approach for large-scale functional enrichment studies, including biological process (BP), molecular function (MF) and cellular component (CC). The Kyoto Encyclopedia of Genes and Genomes (KEGG) is a widely used database that stores information about genomes, biological pathways, diseases, and drugs. GO analysis and KEGG enrichment analysis of DEGs were performed using the clusterProfiler package (Yu et al., 2012), and results with *p* < 0.05 were considered statistically significant.

### 2.7 Gene set enrichment analysis and gene set variation analysis

To investigate the differences in biological processes between different subgroups, based on the integrated GSE34095 and GSE70362 disease samples, we used gene set enrichment analysis (GSEA). GSEA is an algorithm used to evaluate the trend of distribution of genes of a predefined gene set in a table of genes ordered by their phenotypic correlation and thus determine their contribution to the phenotype (Subramanian et al., 2005). Gene set variation analysis (GSVA) is a non-parametric unsupervised analysis method that evaluates the enrichment of gene sets in the microarray nuclear transcriptome by converting the expression matrix of genes between samples into the expression matrix of gene sets between samples (Hänzelmann et al., 2013). This is used to assess whether different pathways are enriched across samples. We downloaded the c2.all.v7.2.symbols.gmt gene set from the MSigDB database for GSEA and GSVA analysis, and results with *p* < 0.05 was considered significantly enriched.

### 2.8 Nomogram construction

We screened genes with area under curve (AUC) > 0.7 by ROC curves, and based on the results of multifactorial analysis, multiple predictors were integrated and assigned according to certain proportions to visualize the interrelationship between each variable on outcome prediction in the form of a graph. We used multifactorial logistic regression to predict the incidence of IDD based on these senescence-related genes and plotted the Nomogram.

### 2.9 Immune cell infiltration analysis

We performed ssGSEA analysis (Supplementary Table S1) on IDD samples based on immune cell markers by GSVA package, estimated the composition and abundance of 28 immune cells, and compared the differences in immune cells between high- and low-risk groups for IDD. The Spearman algorithm was used to assess the infiltration abundance of significant immune cells for correlation analysis with the risk score.

### 2.10 MiRNA/TF-gene network construction

Networkanalyst is an online visual analysis platform for gene expression analysis and meta-analysis (Zhou et al., 2019). We used Networkanalyst to analyze key senescence-related DEGs associated transcription factors (TFs) based on the JASPAR database. Key senescence-related DEGs associated miRNAs were analyzed by the multiMiR package (Ru et al., 2014). Based on the above results, the correlation network was plotted using Cytoscape software (Shannon et al., 2003).

### 2.11 Patient samples

Human degenerative nucleus pulposus tissues were obtained from 6 patients (4 females and 2 males; age 60.33 ± 4.76 years; Grade IV) with degenerative disc disease undergoing surgery. The control samples were taken from 6 patients (3 females and 3 males; age 30.17 ± 7.61 years; Grade Ⅱ) undergoing surgery due to scoliosis or thoracolumbar fracture. The degenerative grade of NP tissues was determined using the MRI-based Pfirrmann grading system (Pfirrmann et al., 2001). Informed consent was obtained from patients preoperatively. This study protocol was approved by the Ethics Committee of the Xi’an Daxing Hospital, affiliated with Yan’an University.

### 2.12 qPCR

Total RNA was extracted using the RNA extraction solution (cat. no. G3013, Servicebio, China), and the RNA concentration and purity were measured using a Nanodrop 2,000 spectrophotometer (Thermo Scientific, United States). The RNA samples were reverse transcribed into cDNA using a reverse transcription kit (cat. no. G3337, Servicebio, China), and the cDNA was used as a template for amplifying the target genes. The reaction was performed with 40 amplification cycles using the following protocol: denaturation at 95°C for 30 s, annealing at 60°C for 30 s, and extension at 72°C for 60 s. Samples were analyzed in triplicate, and the mRNA expression levels were calculated using the 2^−ΔΔCT^ method, with GAPDH serving as the internal reference. The sequences of the primers are listed in Table 1.

**TABLE 1:** Primer sequences used for qPCR.

Gene	Forward primer (5′-3′)	Reverse primer (5′-3′)
GAPDH	GGA​AGC​TTG​TCA​TCA​ATG​GAA​ATC	TGA​TGA​CCC​TTT​TGG​CTC​CC
CABIN1	CAG​CCC​ATT​CCT​TTC​TTC​ACC​T	TCC​TGG​TAG​TCC​GAC​AAA​TCA​ATC
CDKN2B	GAA​TGC​GCG​AGG​AGA​ACA​AG	CAT​CAT​CAT​GAC​CTG​GAT​CGC
CDKN2C	AGG​GGG​GAC​CTA​GAG​CAA​CTT​AC	GGC​AGG​TTC​CCT​TCA​TTA​TCC
CXCL8	CTC​TTG​GCA​GCC​TTC​CTG​ATT​T	GGG​GTG​GAA​AGG​TTT​GGA​GTA​T
H1-5	AGG​CGC​CGC​TAA​AGC​TAA​G	CCT​TCT​TAG​GGC​TCT​TCG​CC
MAP4K4	AGG​CGA​GAA​AGA​TGA​AAC​TGA​G	TGT​TCT​TTC​TGC​TGC​TCA​ATC​C
MAPK11	TCA​GAA​CAC​GCC​CGG​ACA​TA	TGT​CCA​GCA​CCA​GCA​TCC​TT
MDM2	TCA​ATC​AGC​AGG​AAT​CAT​CGG​A	TTG​TGG​CGT​TTT​CTT​TGT​CGT​T
MINK1	CCT​GAA​GGA​GGA​CTG​TAT​CGC	CCA​ATG​AAA​GTG​TTC​CGT​CTG​C
TFDP2	GAT​GGG​AAT​GTC​GTT​TGG​CC	CTT​CTG​CAT​CCA​AGC​ATA​ACC​CT
TINF2	GGC​TCA​CCA​ACC​CAG​GTC​ATA	CTC​TGT​GGC​AGG​CAA​GTC​AA
TNIK	ACT​TTC​ATT​GGA​ACT​CCC​TAC​TGG	TCG​CTG​GCT​GTG​ATT​CTT​TAC​C

### 2.13 HE and Safranin O staining

HE staining (cat. no. G1003, Servicebio, China) and Safranin O staining (cat. no. G1053, Servicebio, China) were performed according to manufacturer’s instructions, and the results were recorded under a microscope (Nikon, Japan).

### 2.14 Fluorescence *in situ* hybridization (FISH)

RNA probes were designed and obtained from Servicebio (Wuhan, China), see Table 2 for probe details. Initially, paraffin slices underwent dewaxing and dehydration. Next, the slides were immersed in a boiling retrieval solution, treated with proteinase K for digestion, and then incubated with prehybridization solution for 1 h at 37°C. Following removal of the prehybridization solution, the slides were exposed to probe hybridization solution in a humidity chamber overnight at 40°C. Subsequently, the slides were washed, signal probe hybridization solution was added, and nuclear counterstaining with DAPI was performed according to the manufacturer’s instructions. Finally, the slices were placed under a fluorescence microscope (Nikon, Japan) for observation and image capture.

**TABLE 2:** Probe sequences used for FISH.

Probe names	Probe sequences (5′-3′)
CDKN2C	GTG​TGC​TTC​ACC​AGG​AAC​TCC​ACC​A, CAA​ATC​CAT​TTT​GTG​CAT​TGA​CGT​T, TAT​TGA​AGA​TTT​GTG​GCT​CCC​CCA​G, TCT​TTC​AAA​TCG​GGA​TTA​GCA​CCT​C, GCA​TCA​TGA​ATG​ACA​GCG​AAA​CCA​G
CXCL8	AAA​TCA​GGA​AGG​CTG​CCA​AGA​GAG​C, AAA​CTT​CTC​CAC​AAC​CCT​CTG​CAC​C, GGG​GTG​GAA​AGG​TTT​GGA​GTA​TGT​C, CGC​AGT​GTG​GTC​CAC​TCT​CAA​TCA​C, GGG​TCC​AGA​CAG​AGC​TCT​CTT​CCA​T
MAP4K4	TGT​AAT​TCT​TCA​CCA​GGC​ACC​CTT​C, ACC​AGC​TCA​AAA​ATC​CCA​GCA​GGA​T, CAC​TCC​AGT​AAC​ATG​GCC​TGC​TCC​T, GTT​CAC​GTT​CCT​GCT​GCC​TTC​TAG​C, GGC​CTC​CTA​TGG​TCA​TGC​TGT​AGA​G
TFDP2	GGA​CTC​CCA​ATC​AGA​ACA​CTT​CCT​G, AAC​GAC​ATT​CCC​ATC​CGC​TTT​AGT​A, GCA​TCC​AAG​CAT​AAC​CCT​TGG​TTT​A, GCA​GCC​AAA​TGG​TTA​TTT​GAA​TTG​G, TGT​AGG​AGA​AGT​TCT​TGC​AGC​TGG​G
TNIK	ATC​TAT​TTC​ATC​CAG​GCT​TCG​AGC​C, ATG​CAA​TCC​ACT​CCT​CTT​TCA​ACG​T, CAC​TTT​ATG​CTG​GTG​CAG​GTG​ACT​C, AGC​TGA​GCA​CTG​ACT​CCA​AAG​TCC​A, AGC​ACC​ACA​AAA​CTC​CAT​CAC​CAA​C

### 2.15 Immunohistochemistry

After deparaffinization and rehydration of paraffin-embedded tissue sections, they were blocked with 3% BSA blocking solution (cat. no. GC305010, Servicebio, China) at room temperature for 30 min. The sections were then incubated overnight at 4°C with primary antibodies: MAP4K4 (Rabbit, cat. no. 55247-1-AP, Proteintech, China), CXCL8 (Rabbit, cat. no. 17038-1-AP, Proteintech, China), TFDP2 (Rabbit, cat. no. 11500-1-AP, Proteintech, China), TNIK (Mouse, cat. no. 67948-1-Ig, Proteintech, China), CDKN2C (Rabbit, cat. no. AF0620, Affinity Biosciences, China). After washing the sections, they were incubated with secondary antibodies (cat. no. GB23303, Servicebio, China) for 50 min. Finally, the sections were rinsed with water, counterstained with hematoxylin, and mounted with a coverslip. The results were examined under an optical microscope (Nikon, Japan).

### 2.16 Statistical analysis

All data calculations and statistical analyses were performed using R software (https://www.r-project.org/, version 4.0.2). For the comparison of two groups of continuous variables were analyzed by Mann-Whitney *U* test (Wilcoxon rank sum test). The cut-off for statistical significance was set at *p* < 0.05.

## 3 Results

### 3.1 Flow chart of the study

**FIGURE 1:**
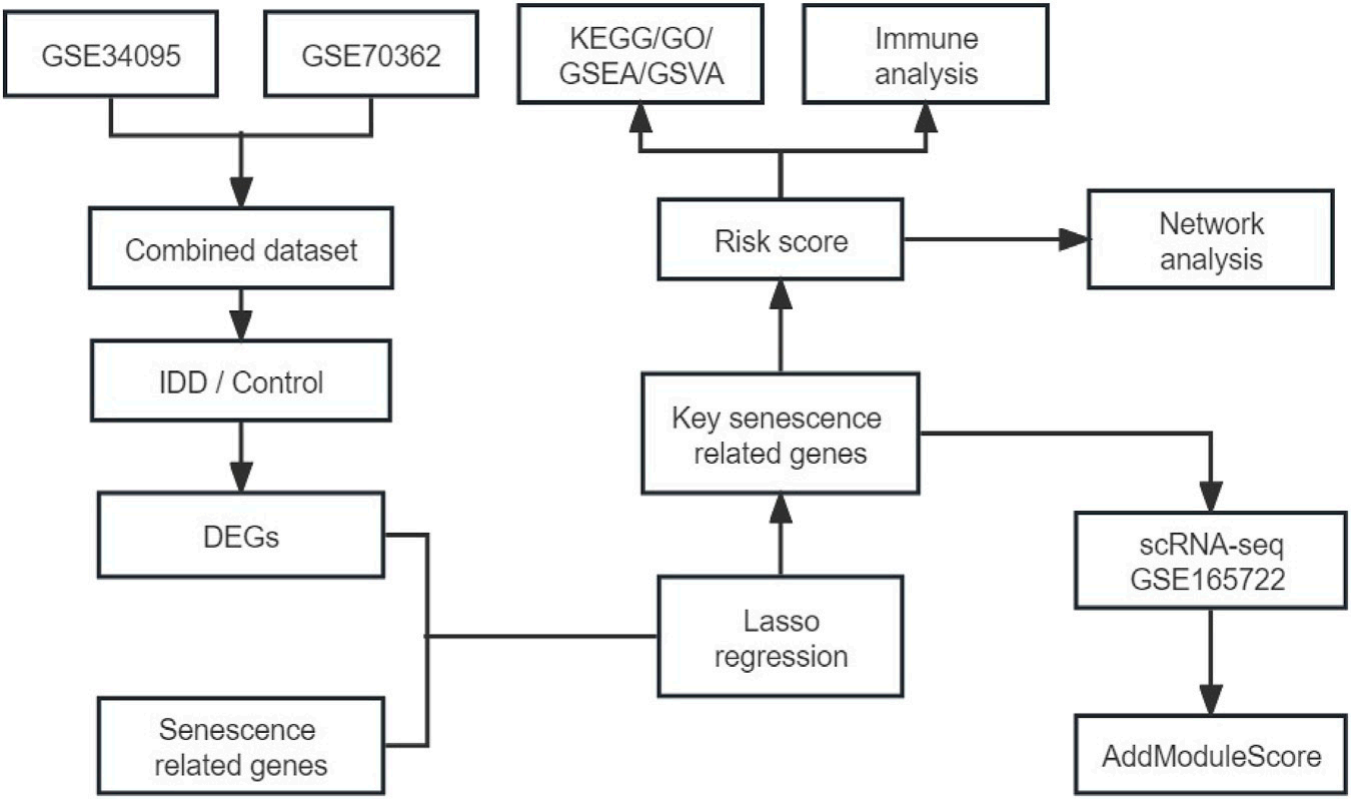
Flow chart of the study.

We merged the GSE34095 and GSE70362 datasets then removed the batch effect. The integrated dataset was used to identify DEGs using the limma package and intersection was taken with senescence-related genes to identify senescence-related DEGs associated with IDD. Key senescence-related DEGs were identified by LASSO regression and risk scores were established based on this. Next, IDD patients were divided into high- and low-risk groups to compare the differences in pathways, function, and immune cell infiltration between the two groups. Finally, in the single-cell dataset GSE165722, key senescence-related DEGs were scored to evaluate their expression in IDD single-cell samples. The flow chart is shown in Figure 1. The GEO datasets are shown in Supplementary Table S2.

### 3.2 Datasets integration and batch effect removal

**FIGURE 2:**
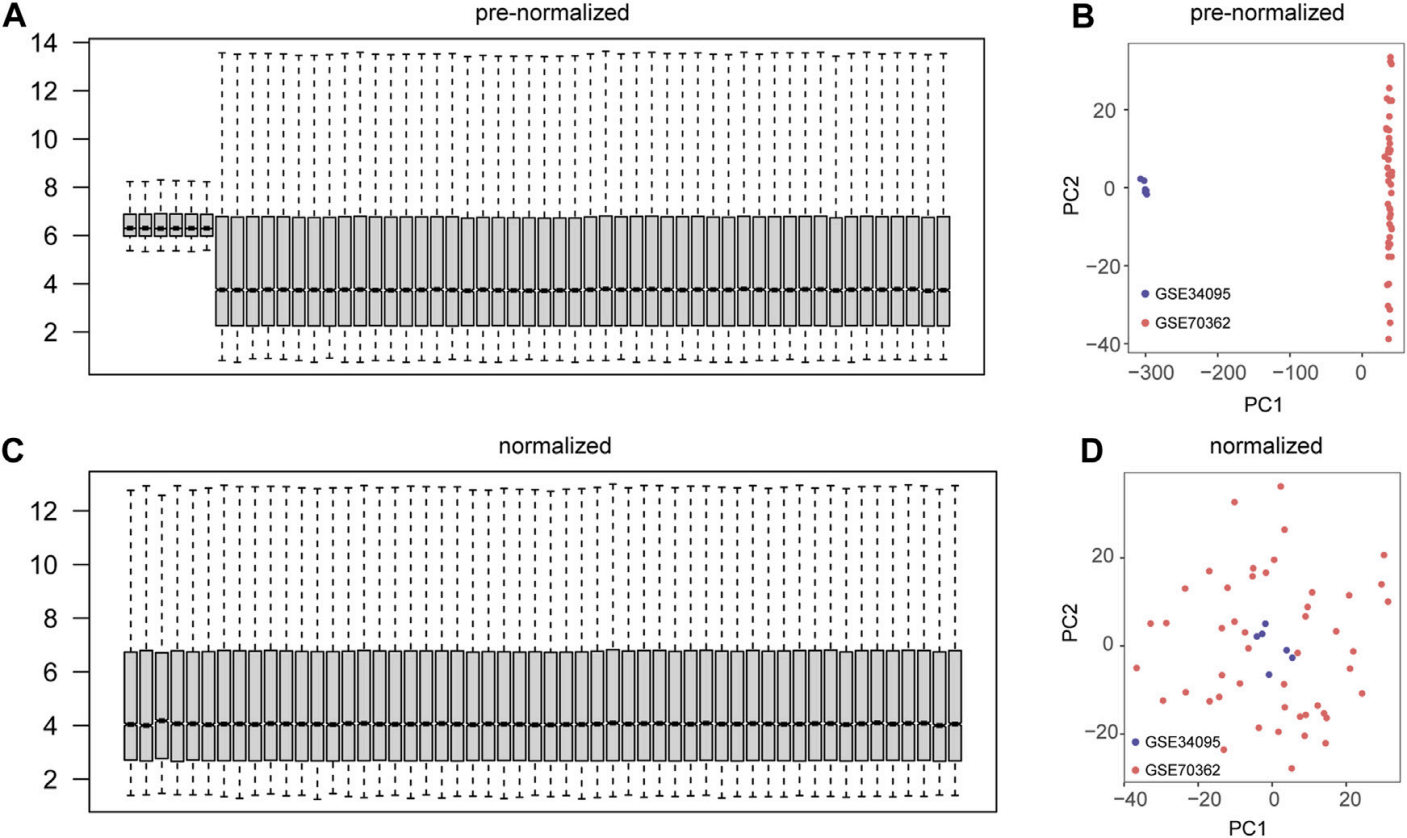
Datasets integration and batch effect removal. **(A)** Box plots of gene expression levels for the combined two datasets (GSE34095 and GSE70362). **(B)** Principal component analysis (PCA) plots of the combined two datasets (GSE34095 and GSE70362). **(C)** Box plots of gene expression levels for the two datasets (GSE34095 and GSE70362) after removal of batch effects. **(D)** PCA plots of two datasets (GSE34095 and GSE70362) after removing batch effects.

We performed a preliminary merge of the GSE34095 and GSE70362 datasets, showing the expression levels of 54 samples with box plots (Figure 2A) and the expression distribution of 54 samples with PCA plots (Figure 2B). We removed batch effects for the combined dataset and showed the expression levels of 54 samples using box plots (Figure 2C) and the expression distribution of 54 samples using PCA plots (Figure 2D). The results showed good correction with no batch effect in the sample.

### 3.3 Identification of senescence-related DEGs

**FIGURE 3:**
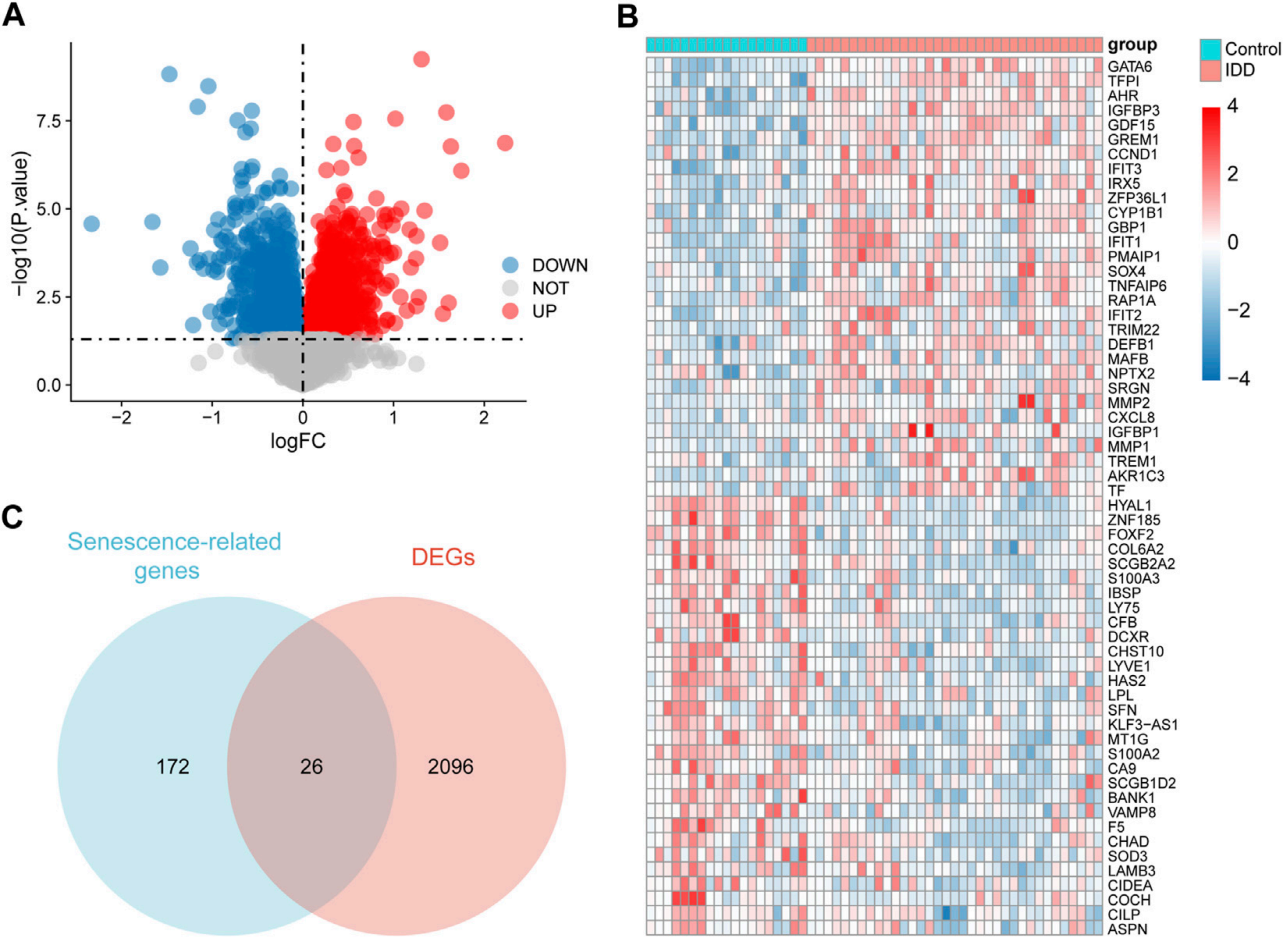
Identification of senescence-related differentially expressed genes (DEGs). **(A)** Volcano diagram of DEGs, red represents upregulation and blue represents downregulation. **(B)** Heat map of DEGs, red represents upregulation and blue represents downregulation. **(C)** Venn diagram of 26 senescence-related DEGs.

A total of 2,122 DEGs were obtained, including 975 upregulated and 1,147 downregulated genes, and all DEGs were displayed in a volcano map (Figure 3A). The 30 genes with the largest log_2_FC and 30 with the smallest log_2_FC were plotted in the heat map (Figure 3B). A total of 26 senescence-related DEGs were obtained from the intersection of senescence-related genes and DEGs (Figure 3C).

### 3.4 Lasso regression to establish senescence-related risk scores

**FIGURE 4:**
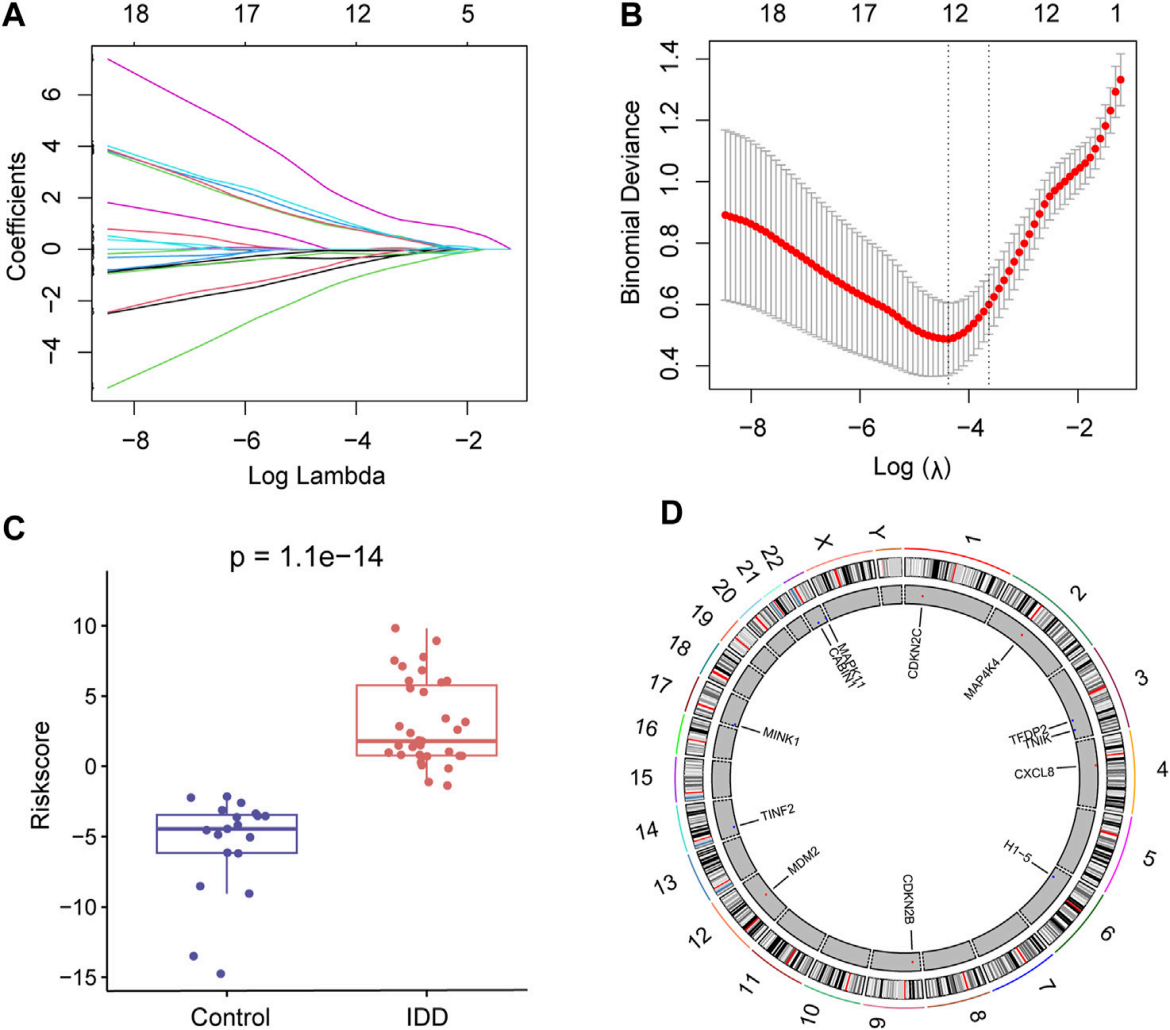
Lasso regression analysis. **(A)** Lasso regression analysis based on 26 senescence-related differentially expressed genes (DEGs). **(B)** Lasso regression analysis obtained 12 key senescence-related DEGs. **(C)** Box plot showing risk scores for the intervertebral disc degeneration (IDD) and control groups. Red represents the IDD group and blue represents the control group. **(D)** Chromosome map showing the chromosomal localization of these 12 key senescence-related DEGs.

To further identify key senescence-related DEGs for IDD, we performed LASSO regression analysis based on 26 senescence-related DEGs (Figure 4A) and obtained 12 key senescence-related DEGs, including, *CABIN1*, *CDKN2B*, *CDKN2C*, *CXCL8*, *H1-5*, *MAP4K4*, *MAPK11*, *MDM2*, *MINK1*, *TFDP2*, *TINF2*, *and TNIK* (Figure 4B). Based on these key genes we developed a diagnostic model. Risk score = −0.747× *CABIN1* + 0.887× *CDKN2B*+ 1.06× *CDKN2C* + 1.21× *CXCL8*–0.0238× *H1-5* + *MAP4K4*×2.19–0.356× *MAPK11* + 0.935× *MDM2*–0.148× *MINK1*–0.0520× *TFDP2*–0.567× *TINF2*–1.36× *TNIK*. This risk score could distinguish well between the control and disease groups (Figure 4C). Subsequently, used chromosome mapping, we presented the chromosomal localization of these 12 genes (Figure 4D).

### 3.5 TFs and miRNAs prediction of key senescence-related DEGs

**FIGURE 5:**
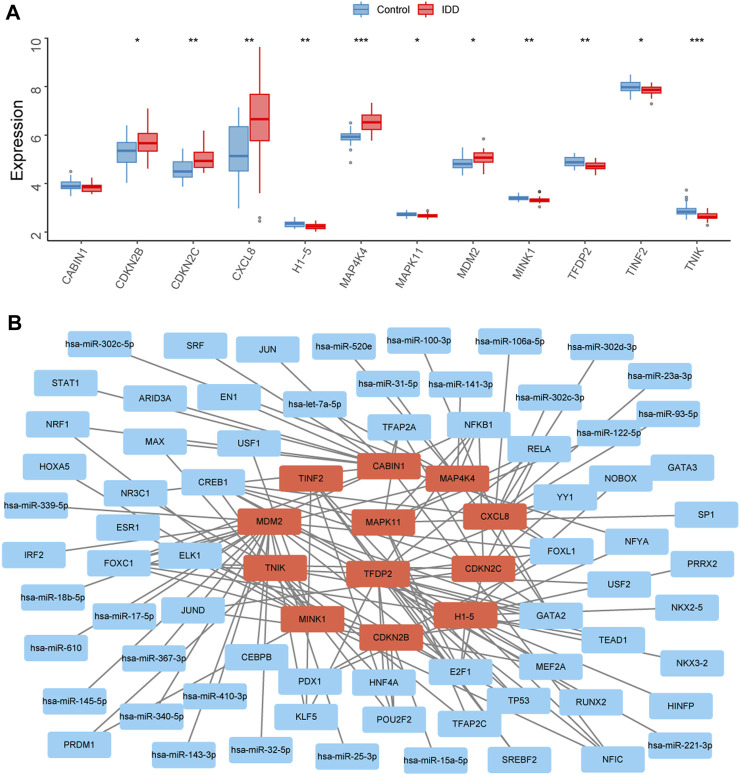
Transcription factors (TFs) and miRNAs prediction of key senescence-related differentially expressed genes (DEGs). **(A)** Box plots showing the expression levels of CABIN1, CDKN2B, CDKN2C, CXCL8, H1-5, MAP4K4, MAPK11, MDM2, MINK1, TFDP2, TINF2, and TNIK in the intervertebral disc degeneration (IDD) and control groups. Red represents the IDD group, blue represents the control group. *p < 0.05, **p < 0.01, ***p < 0.001. **(B)** Interaction network diagram showing 25 associated miRNAs and 45 TFs for 12 key senescence-related DEGs.

The expression levels of the 12 key senescence-related DEGs in the IDD and control groups were demonstrated by box plots. Five of these genes were significantly highly expressed in IDD: *CDKN2B* (*p* < 0.05), *CDKN2C* (*p* < 0.01), *CXCL8* (*p* < 0.01), *MAP4K4* (*p* < 0.001), and *MDM2* (*p* < 0.05). Six genes were significantly low expressed in IDD: *H1-5* (*p* < 0.01), *MAPK11* (*p* < 0.05), *MINK1* (*p* < 0.01), *TFDP2* (*p* < 0.01), *TINF2* (*p* < 0.05), and *TNIK* (*p* < 0.05) (Figure 5A). A total of 25 relevant miRNAs and 45 TFs were obtained through the JASPAR database and multiMiR, and the network was mapped (Figure 5B).

### 3.6 Validation of key senescence-related DEGs

**FIGURE 6:**
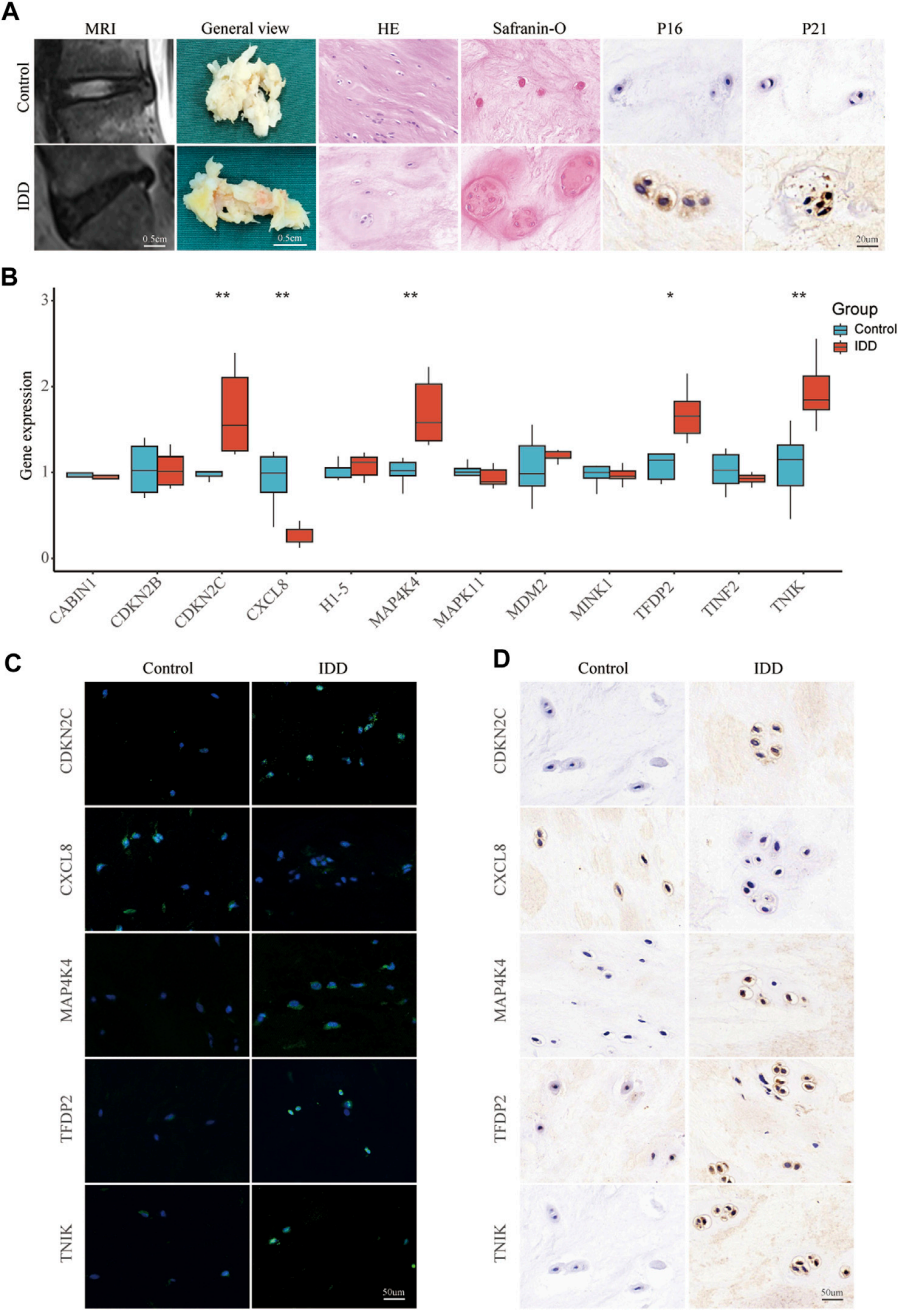
Validating key senescence-related DEGs. **(A)** Representative images were obtained using magnetic resonance imaging, and NP tissues obtained from patients with intervertebral disc degeneration were stained with HE, Safranin O, and immunohistochemical staining for P16 and P21. **(B)** qPCR validate the expression levels of CABIN1, CDKN2B, CDKN2C, CXCL8, H1-5, MAP4K4, MAPK11, MDM2, MINK1, TFDP2, TINF2, and TNIK in IDD and control nucleus pulposus tissue. Red represents the IDD group, blue represents the control group. *p < 0.05, **p < 0.01. Representative images of CDKN2C, MAP4K4, TFDP2, TNIK, and CXCL8 expression in NP tissue detected by FISH **(C)** and Immunohistochemistry **(D)**.

To screen high-quality NP tissues for subsequent experiments, we collected control (grade II) and IDD (grade IV) NP tissues using the MRI-based Pfirrmann grading system. Grade II NP tissues exhibited high water content and appeared as high signal intensity in T2-weighted MRI, presenting a more gelatinous texture under macroscopic observation. In contrast, grade IV NP tissues showed low signal intensity in T2-weighted MRI and varying degrees of fibrotic changes when observed macroscopically (Figure 6A). HE staining revealed distinct characteristics of the tissues associated with increasing IDD grade. Grade II NP cells displayed a uniformly dark-stained extracellular matrix (ECM) with a few small vacuolated cells. Conversely, grade IV cells exhibited a uniformly light-stained ECM with heavily aggregated and large vacuolated cells (Figure 6A). Safranin O staining indicated reduced levels of proteoglycan and collagen fiber in the ECM of grade IV tissues (Figure 6A). Immunohistochemical staining was employed to detect the expression of P16 and P21. The results revealed a higher expression of P16 and P21 in the IDD group, suggesting increased cellular senescence (Figure 6A). In the qPCR results, the expression levels of CDKN2C (*p* < 0.01), MAP4K4 (*p* < 0.01), TFDP2 (*p* < 0.05), and TNIK (*p* < 0.01) in the IDD group were significantly higher compared to the control group. However, CXCL8 showed a significant decrease (*p* < 0.01) in the IDD group (Figure 6B). The FISH and Immunohistochemistry results were generally consistent with the qPCR (Figures 6C, D).

### 3.7 ROC curve

**FIGURE 7:**
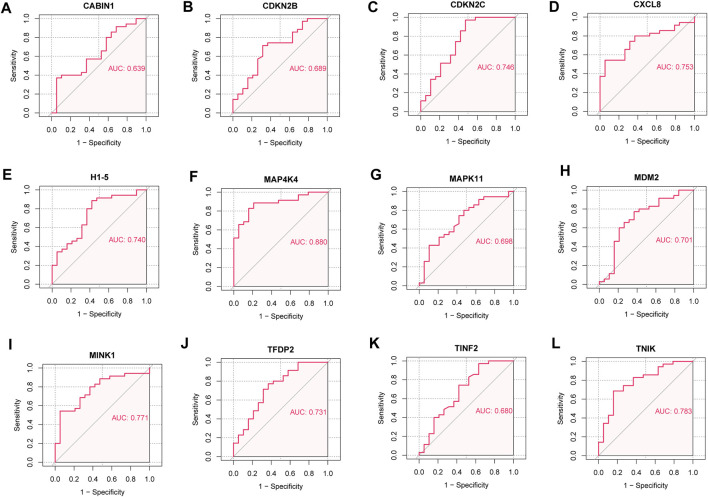
Receiver operating characteristic (ROC) curve. **(A)** ROC curve of CABIN1. **(B)** ROC curves of CDKN2B. **(C)** ROC curves of CDKN2C. **(D)** ROC curve of CXCL8. **(E)** ROC curves of H1-5. **(F)** ROC curve of MAP4K4. **(G)** ROC curve of MAPK11. **(H)** ROC curves of MDM2. **(I)** ROC curve of MINK1. **(J)** ROC curve of TFDP2. **(K)** ROC curve of TINF2. **(L)** ROC curves of TNIK.

We analyzed the ROC curves of key senescence-related DEGs for identifying IDD and controls, and labeled the corresponding AUC. AUC of *CABIN1* = 0.639 (Figure 7A), AUC of *CDKN2B* = 0.689 (Figure 7B), AUC of *CDKN2C* = 0.746 (Figure 7C), AUC of *CXCL8* = 0.753 (Figure 7D), AUC of *H1-5* = 0.740 (Figure 7E), AUC of *MAP4K4* = 0.880 (Figure 7F), AUC of AUC of *MAPK11* = 0.698 (Figure 7G), AUC of *MDM2* = 0.701 (Figure 7H), AUC of *MINK1* = 0.771 (Figure 7I), *AUC* of *TFDP2* = 0.731 (Figure 7J), AUC of *TINF2* = 0.680 (Figure 7K) and AUC of *TNIK* = 0.783 (Figure 7L).

### 3.8 Nomogram

**FIGURE 8:**
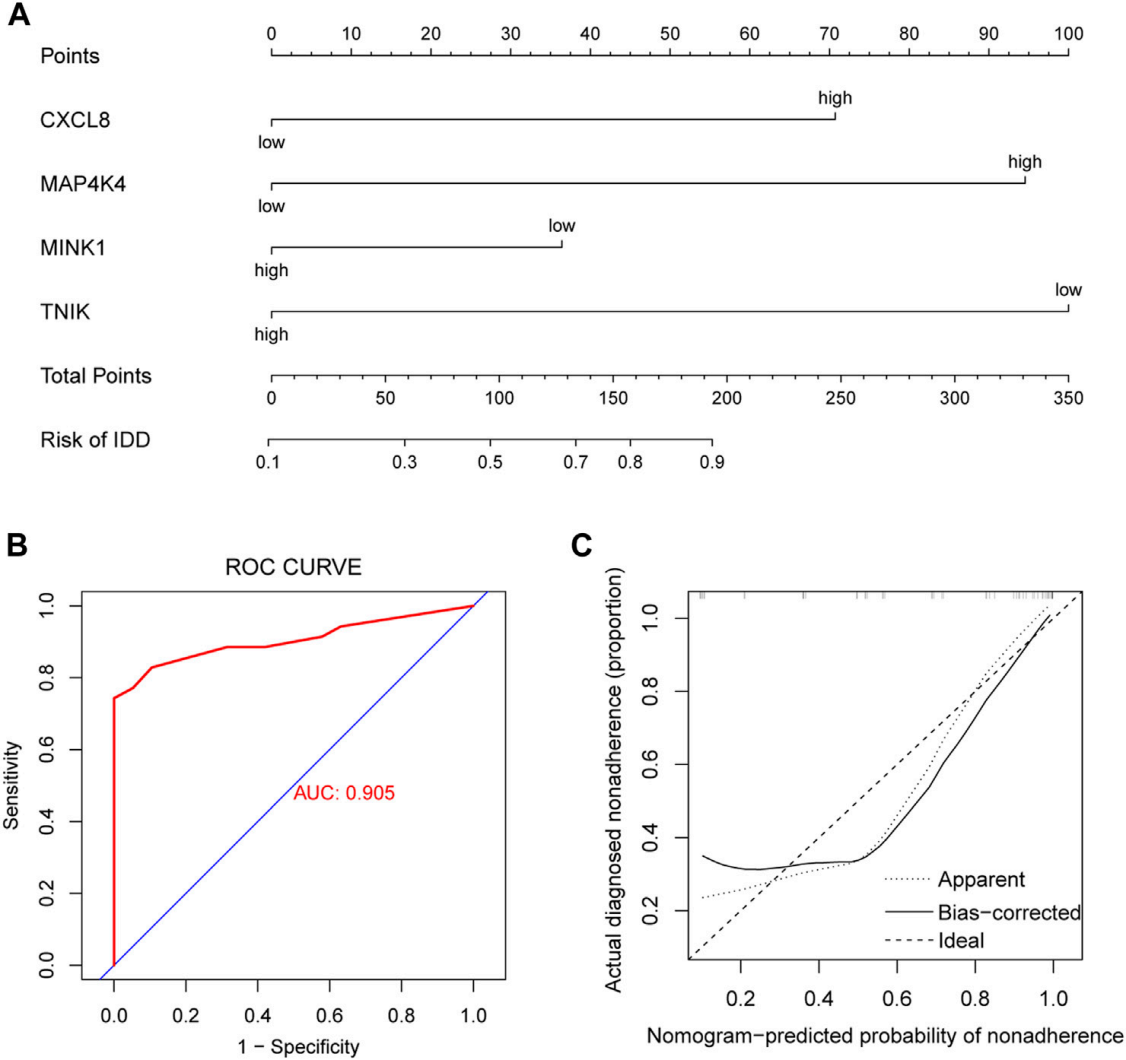
Clinical prediction model. **(A)** Nomogram plots based on CXCL8, MAP4K4, MINK1, and TNIK expression to identify intervertebral disc degeneration (IDD) and controls. **(B)** Receiver operating characteristic (ROC) curves of clinical prediction models. **(C)** Calibration plot of the clinical prediction model.

The incidence of IDD was predicted by constructing models using genes with AUC >0.75 (*CXCL8*, *MAP4K4*, *MINK1*, and *TNIK*), performing multifactorial logistic regression and plotting nomogram (Figure 8A), and the model was evaluated. We plotted the ROC curve (AUC = 0.905) (Figure 8B) and the cxalibration plot of the prediction model, suggesting that the prediction model has good predictive power for IDD (Figure 8C).

### 3.9 GO and KEGG enrichment analysis

We identified 2,368 DEGs in the high- and low-risk groups for IDD and performed GO and KEGG enrichment analyses. These DEGs mainly affect biological processes such as cellular response to chemical stress, extrinsic apoptotic signaling pathway, and activation of protein kinase activity (Supplementary Figure S1A), molecular functions such as protein heterodimerization activity, transcription coactivator activity and small GTPase binding (Supplementary Figure S1B) and cellular components such as proteasome complex, endopeptidase complex, and proteasome accessory complex (Supplementary Figure S1C). Pathways such as pathways of neurodegeneration−multiple diseases, amyotrophic lateral sclerosis, and Alzheimer’s disease (Supplementary Figure S1D). Detailed information about the significantly enriched GO and KEGG categories is provided in Supplementary Tables S3, S4.

### 3.10 GSEA and GSVA enrichment analysis

**FIGURE 9:**
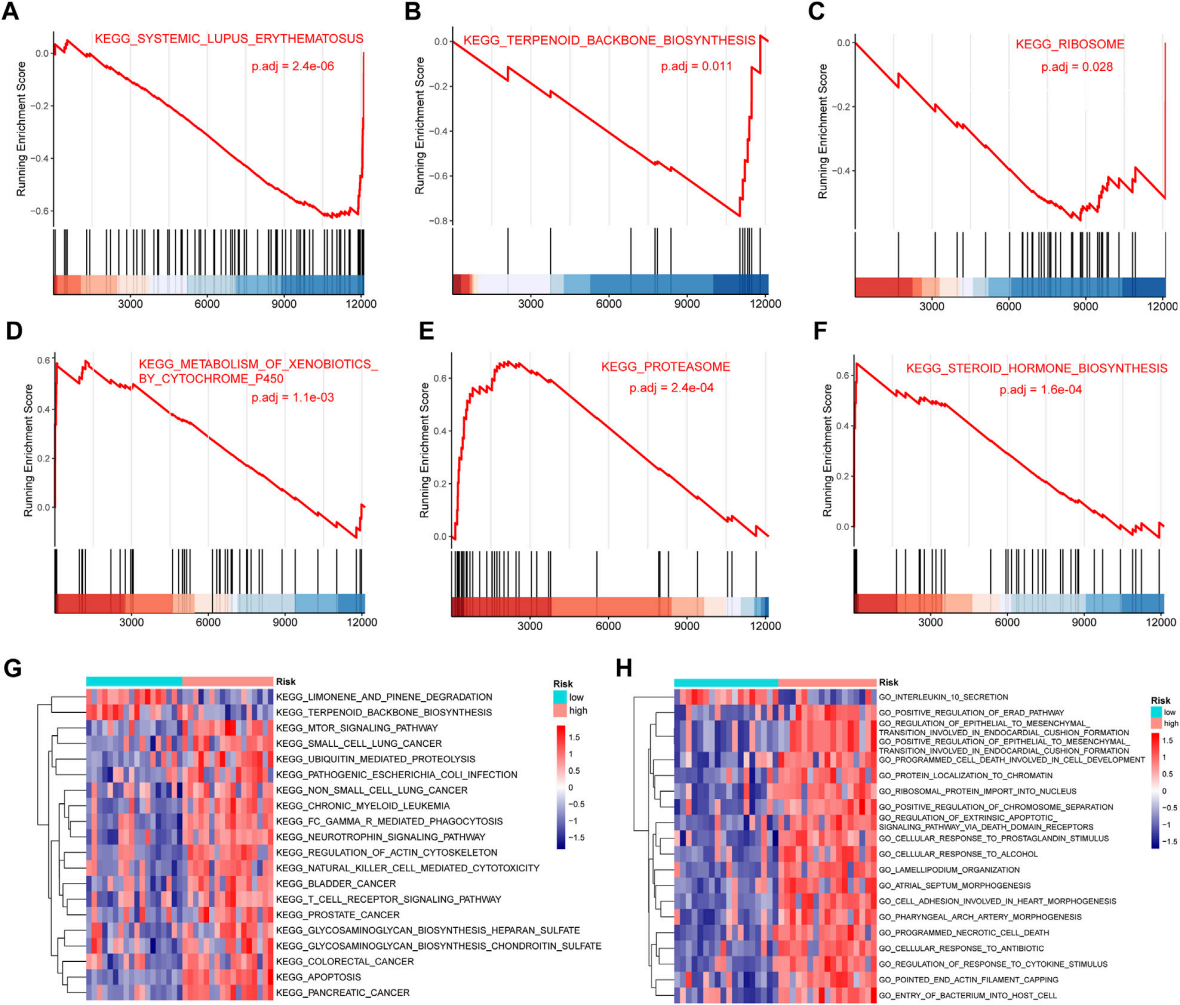
Gene set enrichment analysis (GSEA) and gene set variation analysis (GSVA). **(A–C)** KEGG SYSTEMIC LUPUS ERYTHEMATOSUS **(A)**, KEGG TERPENOID BACKBONE BIOSYNTHESIS **(B)**, KEGG RIBOSOME **(C)** were significantly enriched in the low-risk group. **(D–F)** KEGG CELL CYCLE **(D)**, KEGG DNA REPLICATION **(E)**, KEGG MISMATCH REPAIR **(F)** were significantly enriched in the high-risk group. **(G)** Heat map showing the 20 most significantly different Kyoto Encylopedia of Genes and Genomes (KEGG) pathways in the high- and low-risk groups. Red and blue represent pathways with high and low expression in that group, respectively. **(H)** Heat map showing the 20 most significantly different Gene Ontology (GO)-related functions in the high- and low-risk groups. Red represents pathways with high expression in that group, blue represents pathways with low expression in that group.

We performed a GSEA analysis (Supplementary Table S5) to further analyze the differences between the high- and low-risk groups for IDD. Among them, KEGG SYSTEMIC LUPUS ERYTHEMATOSUS (P.adj = 2.4e-06) (Figure 9A), KEGG TERPENOID BACKBONE BIOSYNTHESIS (P.adj = 0.011) (Figure 9B) and KEGG RIBOSOME (Figure 9C) (P.adj = 0.028) were significantly enriched in the low-risk group. KEGG METABOLISM OF XENOBIOTICS BY CYTOCHROME P450 (P.adj = 1.1e-03) (Figure 9D), KEGG PROTEASOME (P.adj = 2.4e-04) (Figure 9E), KEGG STEROID HORMONE BIOSYNTHESIS (P.adj = 1.6e-04) (Figure 9F) were significantly enriched in the high-risk group. We also performed GSVA analysis and drew a heat map showing the 20 most relevant KEGG pathways (Figure 9G). The pathways KEGG LIMONENE AND PINENE DEGRADATION and KEGG TERPENOID BACKBONE BIOSYNTHESIS were significantly enriched in the low-risk group and the pathways KEGG MTOR SIGNALING PATHWAY and KEGG UBIQUITIN MEDIATED PROTEOLYSIS were significantly enriched in the high-risk group. Figure 9H shows the 20 most relevant GO functions, GO INTERLEUKIN 10 SECRETION was significantly enriched in the low-risk group, GO POSITIVE REGULATION OF ERAD PATHWAY and GO PROTEIN LOCALIZATION TO CHROMATIN were significantly enriched in the high-risk group.

### 3.11 Immune infiltration analysis

**FIGURE 10:**
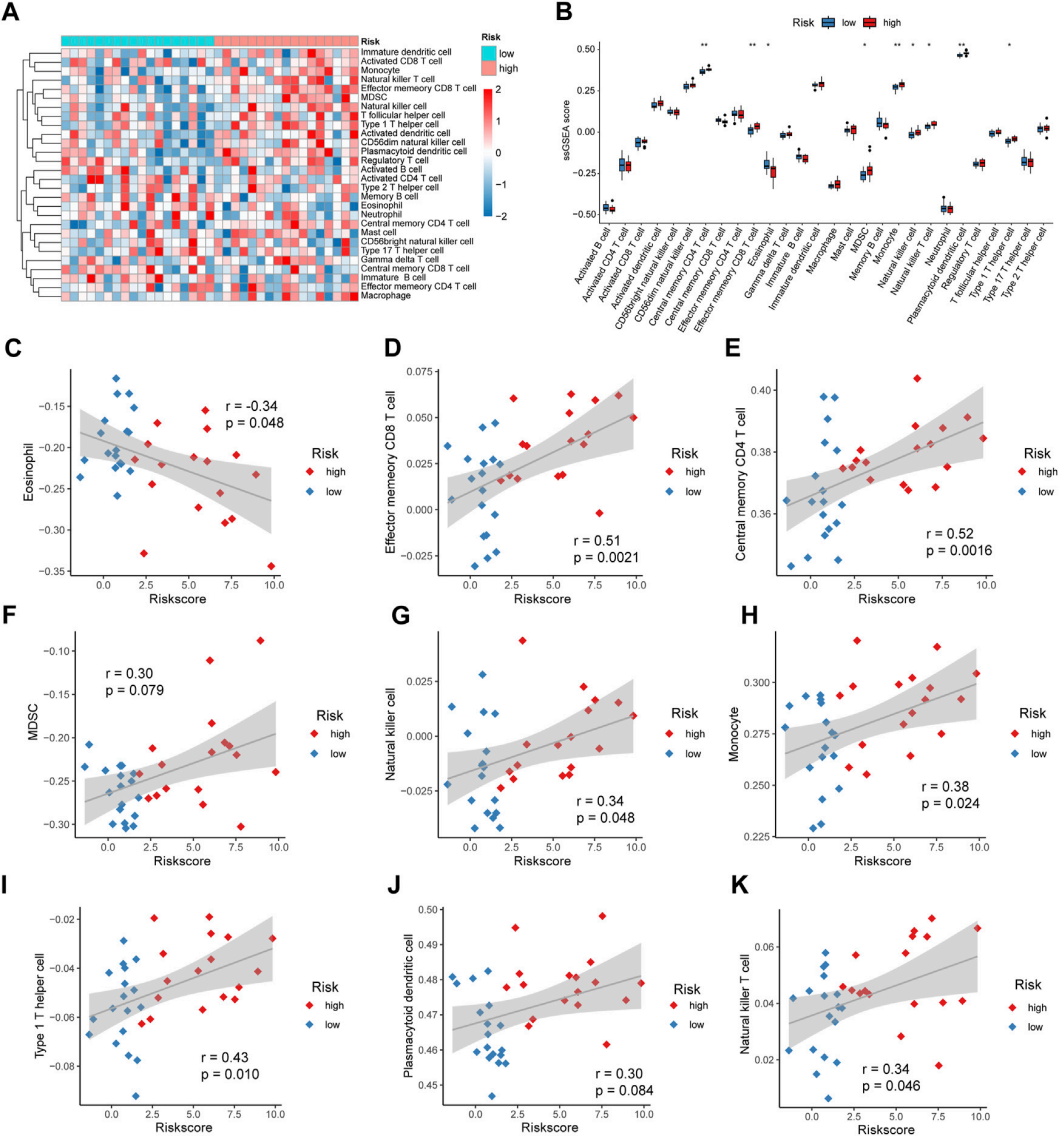
Risk score and immune cell infiltration. **(A)** Heat map showing the abundance of 28 immune cell infiltrates in intervertebral disc degeneration (IDD). **(B)** Box plot showing the difference in immune infiltration levels between high- and low-risk groups, with blue and red representing low- and high-risk groups, respectively. **(C)** Correlation analysis of eosinophil and risk scores. **(D)** Correlation analysis of effector memory CD8 T cells and risk scores. **(E)** Correlation analysis of central memory CD4 T cells and risk scores. **(F)** Correlation analysis between myeloid-derived suppressor cells (MDSCs) and risk score. **(G)** Correlation analysis between natural killer cells and risk score. **(H)** Correlation analysis of monocytes and risk score. **(I)** Correlation analysis between Type 1 T helper cells and risk score. **(J)** Correlation analysis between plasmacytoid dendritic cells and risk score. **(K)** Correlation analysis of natural killer T cells and risk score.

We used ssGSEA to calculate the levels of 28 immune cell infiltrates in patients in the high- and low-risk groups for IDD, plotted a heat map for display (Figure 10A), and plotted a box plot comparing the differences in immune cell infiltration levels between patients in the high-risk and low-risk groups. Nine immune cells were significantly different, with eosinophils being significantly elevated in patients in the low-risk group (*p* < 0.05); Effector memory CD8 T, central memory CD4 T, myeloid-derived suppressor cells (MDSCs), natural killer, monocyte, type 1 T helper cell, plasmacytoid dendritic cell, and natural killer T cells were significantly elevated in patients in the high-risk group (*p* < 0.05) (Figure 10B). Subsequently, we calculated the correlation between the abundance of immune cell infiltration and the risk score. Eosinophils were negatively correlated with the risk score (cor = −0.34, *p* = 0.048) (Figure 10C); Effector memory CD8 T cells were positively correlated with risk score (cor = 0.51, *p* = 0.0021) (Figure 10D); Central memory CD4 T cells were positively correlated with risk score (cor = 0.52, *p* = 0.0016) (Figure 10E), MDSCs were positively correlated with risk score (cor = 0.30, *p* = 0.079) (Figure 10F); Natural killer cells were positively correlated with risk score (cor = 0.34, *p* = 0.048) (Figure 10G); Monocytes were positively correlated with risk score (cor = 0.38, *p* = 0.042) (Figure 10H), Type 1 T helper cells were positively correlated with risk score (cor = 0.43, *p* = 0.010) (Figure 10I); Plasmacytoid dendritic cells were positively correlated with risk score (cor = 0.30, *p* = 0.084) (Figure 10J); Natural killer T cells were positively correlated with risk score (cor = 0.34, *p* = 0.046) (Figure 10K).

### 3.12 GSE165722 single-cell analysis

**FIGURE 11:**
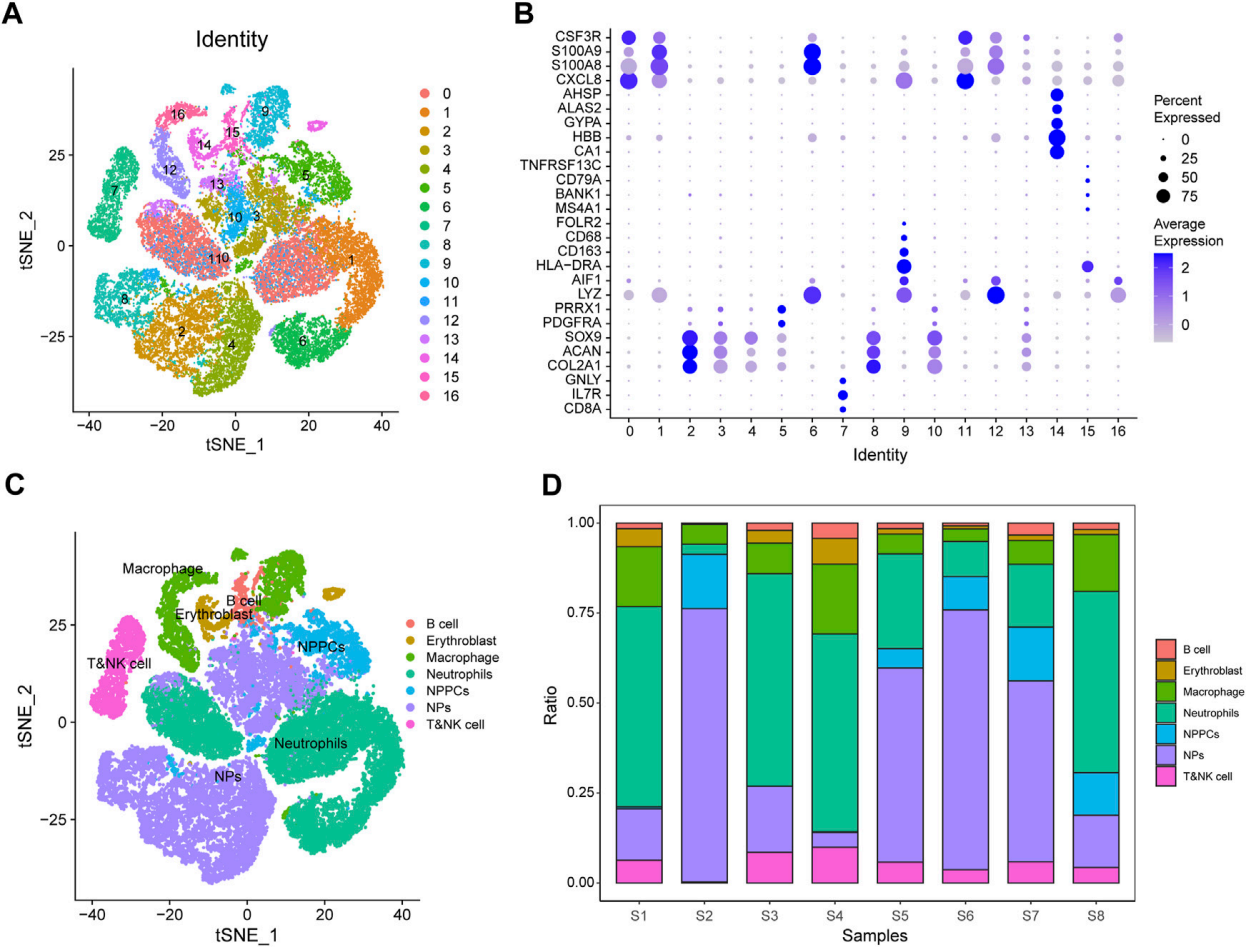
GSE165722 single-cell sequencing analysis. **(A)** Reduced-dimensional cluster analysis divides cells into 16 clusters and presents them with t-distributed stochastic neighbor embedding (t-SNE) plots. **(B)** Cellular annotation heat map showing the expression levels of markers for 16 clusters. **(C)** Annotation of 16 clusters containing 7 cell types: B cells, nucleated erythrocytes, macrophages, neutrophils, myeloid cells, myeloid progenitor cells, and T or NK cells. **(D)** Histogram of cell proportions, where the horizontal coordinates represent cell proportions and the vertical coordinates represent sample names.

We performed single-cell data analysis on eight IDD samples from GSE165722 and divided the cell population into 16 clusters using tSNE analysis (Figure 11A), and each cluster was annotated by markers of the cells and plotted in bubble plots for display (Figure 11B). The cell cluster was divided into B cells, nucleated erythrocytes, macrophages, neutrophils, myeloid cells, myeloid progenitor cells and T or NK cells according to the results of cell annotation (Figure 11C), and a proportional histogram was drawn to show the proportion of cells in these eight IDD samples (Figure 11D).

### 3.13 GSE165722-based senescence scoring

**FIGURE 12:**
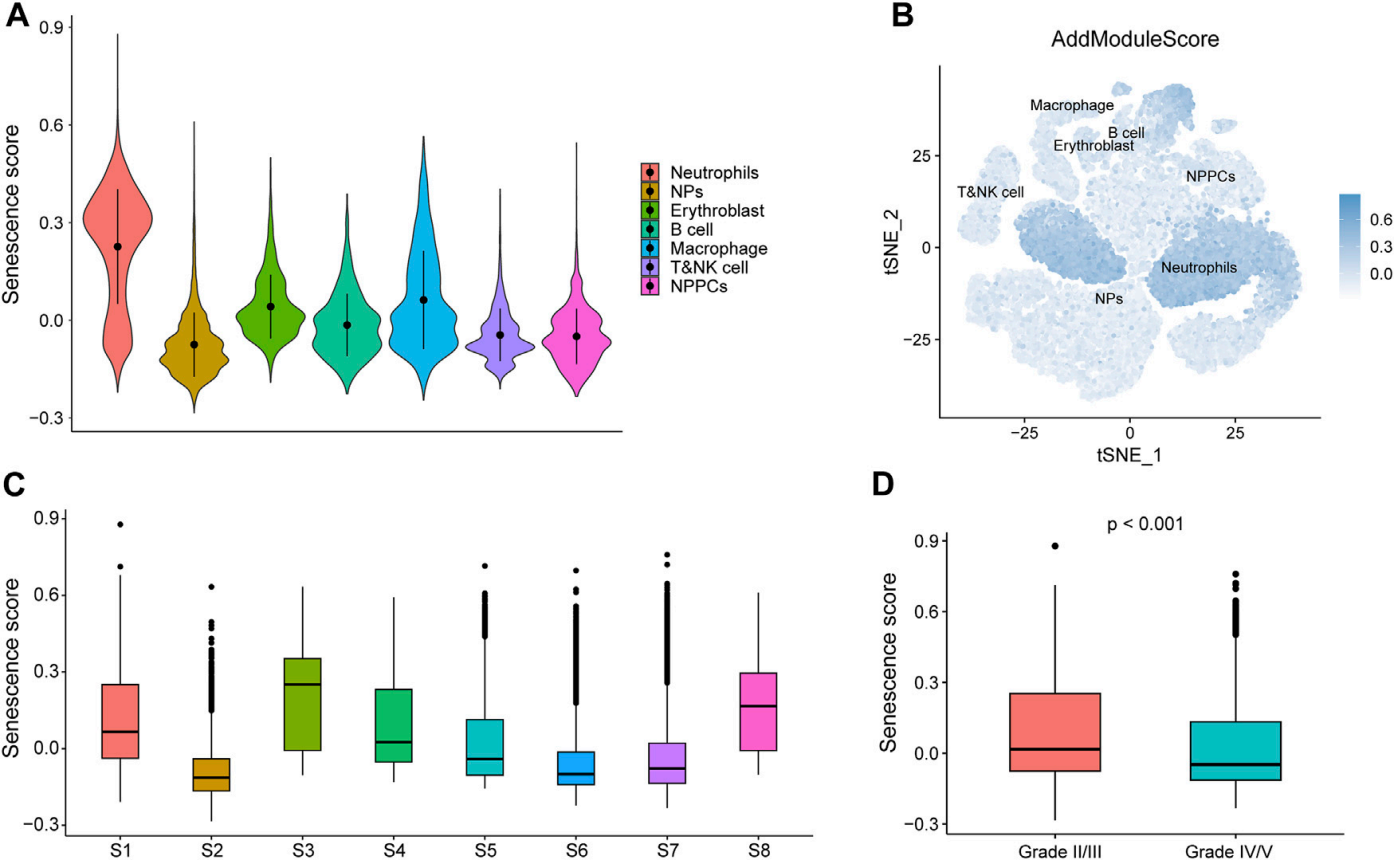
Senescence score based GSE165722. **(A)** AddModuleScore calculated senescence score. Violin diagram of different cell expression. **(B)** Senescence score distributed stochastic neighbor embedding (t-SNE). Blue represents high scores, while white represents low scores. **(C)** Box plots showing the senescence scores of different samples in GSE165722. **(D)** Box plots showing senescence score for different grades of intervertebral disc degeneration (IDD), with red representing grade II/II and blue representing grade IV/V.

Senescence scores were calculated for individual cells (B cells, nucleated red blood cells, macrophages, neutrophils, myeloid cells, myeloid progenitor cells, and T or NK cells) based on key senescence-related DEGs (*CABIN1*, *CDKN2B*, *CDKN2C*, *CXCL8*, *H1-5*, *MAP4K4*, *MAPK11*, *MDM2*, *MINK1*, *TFDP2*, *TINF2*, and *TNIK*) using the AddModuleScore function (Figure 12A). Using the calculated senescence score to plot tSNE, we found that the senescence score was significantly highly expressed in neutrophils (Figure 12B). We show the senescence scores of these eight IDD samples using box plots (Figure 12C) and that IDD patients with IV/V classification have lower senescence scores relative to IDD patients with II/III classification. This result suggests that these genes may play a role in neutrophils in IDD (Figure 12D).

## 4 Discussion

IDD is the most common cause of lower back pain (Cheng et al., 2018). The pathophysiological mechanisms of IDD are complex and not fully understood. IDD is closely associated with senescence. Therefore, it is crucial to explore the key senescence-related genes and their pathogenic mechanisms to provide an experimental and theoretical basis for the repair of IDD.

In this study, 12 key senescence-related DEGs were identified. A risk score was established based on this, which could distinguish well between the control and IDD groups. In addition, a nomogram for predicting IDD prevalence was constructed by selecting genes with AUC >0.75 (*CXCL8*, *MAP4K4*, *MINK1*, and *TNIK*). The ROC curve and the calibration plot suggest that the nomogram has good predictive power for IDD. Calcineurin upregulation was observed in hypoxic nucleus pulposus cells (Huang et al., 2020). CABIN1, a natural calcineurin antagonist, is speculated to play a significant role in IDD. CDKN2B and CDKN2C regulate the cell cycle and are closely linked to cellular senescence (Gagrica et al., 2012; Park et al., 2023). These findings suggest that CDKN2B and CDKN2C could be potential targets for IDD. However, further experiments are needed to confirm the above hypothesis. CXCL8 plays a role in neurogenic pain caused by disc herniation, with its expression correlating to the severity of degenerative tissue changes in the nucleus pulposus (Phillips et al., 2013; Pedersen et al., 2015). MDM2 is highly expressed in degenerated intervertebral discs (Wang et al., 2021). Our study identified significant upregulation of CXCL8 and MDM2 expression in IDD, consistent with previous findings. Prior research demonstrates significant overexpression of MAPK11 in IDD (Yang et al., 2016). Conversely, our study suggests downregulation of MAPK11. Further clinical samples and cellular experiments are required to validate MAPK11 expression in IDD. No prior research has examined the role of H1-5 in IDD. Our study discovered low expression levels of H1-5, highlighting its potential as a promising molecular target for IDD. miR-547-3p regulates inflammatory cytokines by targeting MAP4K4, alleviating neuropathic pain from lumbar disc herniation (Yao et al., 2022). MINK1 and TNIK are associated with senescence, suggesting their potential role in IDD (Reizis, 2019; Yu et al., 2020). Our findings demonstrate downregulation of TFDP2 and TINF2 in IDD. However, their roles in IDD have not been reported and require further investigation.

To further investigate the biological function and pathway differences between patients at different risks, we performed functional and pathway enrichment analysis. The results of this study showed that the two groups of patients differed mainly in cellular response to chemical stress, extrinsic apoptotic signaling pathway, activation of protein kinase activity, and transcription coactivator activity. Oxidative stress has been confirmed as a significant contributor to IDD, with excessive reactive oxygen species causing cellular oxidation and chemical stress, leading to apoptosis of functional disc cells and hastening degeneration (Lin et al., 2020). Protein kinases are critical regulators of cellular function, governing multiple intracellular signaling pathways to maintain normal physiological cell activity (Kupka et al., 2020; Du et al., 2022). DNA-binding transcription factors interact with co-activator complexes to enhance gene expression (Dietrich et al., 2017). Mechanism-sensitive transcriptional coactivators MRTF-A and YAP/TAZ play a role in regulating the phenotype of nucleus pulposus cells through cell shape modulation (Fearing et al., 2019). IL-10 secretion was prominently enriched in the low-risk group, while the high-risk group exhibited significant enrichment in the mTOR pathway and positive regulation of ERAD pathways. Previous research has indicated that IL-10 delays IDD by inhibiting the p38 MAPK pathway, suggesting its protective role in IDD, consistent with our study findings (Ge et al., 2020). ERAD is the primary mechanism for clearing misfolded proteins from the endoplasmic reticulum and has potential as a target for IDD treatment (Wen et al., 2021). Recent studies demonstrate the involvement of mTOR signaling in regulating intervertebral disc cell functions like oxidative stress, inflammation, cellular senescence, and apoptosis (Chen et al., 2023). Further investigation is warranted to explore the potential of targeting mTOR signaling and promoting ERAD pathways as molecular interventions for IDD.

Although there have been some reports on IDD and the immune microenvironment (Saberi et al., 2021; Wang et al., 2021), the relationship between these immune cells and genes remains unknown. High eosinophil levels were found in the low-risk group, with a negative correlation between eosinophil infiltration and the risk score. Effector memory CD8 T, central memory CD4 T, MDSC, natural killer cells, monocyte, type 1 T helper, plasmacytoid dendritic, and natural killer T cells were significantly elevated in the high-risk group of patients, and their infiltration abundance was all positively correlated with the risk score. We discovered that eosinophil abundance is protective in IDD. However, additional research on eosinophils in IDD is required to confirm their role. Plasmacytoid dendritic cells (pDCs) are implicated in IFN-producing diseases (Li et al., 2019). However, the role of pDCs in IDD development has not been reported. Our study found significantly elevated pDC levels in the high-risk group, highlighting the need for further investigation on the pDC-IDD interaction. Studies have shown increased infiltration of CD8^+^ and CD4^+^ T cells in IDD, leading to exacerbation of the inflammatory response (Li et al., 2020; Lan et al., 2022). MDSCs were significantly enriched in severely degenerated nucleus pulposus tissue (Tu et al., 2022). *In vitro* assays demonstrated that NK cells and macrophages have immune function in the early stages of IDD (Stich et al., 2015). There is evidence of monocyte infiltration occurring during the early degenerative stages (Li et al., 2023). Research has shown that in IDD, activated T cells predominantly differentiate into Th1, Th2, and Th17 subsets, with Th1 regulating macrophage involvement in IDD (Francisco et al., 2022). The above studies suggest that patients with IDD of different degenerative periods and degrees have different patterns of immune cell infiltration, which is supported by our findings. These results further deepen the understanding that immune cell infiltration has on different functions in patients with different risks of IDD.

Senescent cells form a senescence associated secretory phenotype (SASP), which induces inflammation, recruits immune cells (such as macrophages, natural killer cells, and neutrophils), and spreads senescence to other cells, thus exerting long-range effects on surrounding cells and tissues (Kale et al., 2020). Through single cell sequencing analysis, we found that the senescence score was significantly high in neutrophils. Neutrophils are typically the first immune cells to arrive at the site of inflammation and respond to bacterial infections. IL-8, as a major SASP cytokine, attracts neutrophils and stimulates their release of antimicrobial granules (Prata et al., 2018). Lagnado et al. (2021) showed that neutrophils can trigger senescence in neighboring cells by delivering reactive oxygen species. Neutrophils show a strong correlation with cellular senescence, indicating potential benefits in countering neutrophil-induced senescence for aging and age-related diseases. This aligns with our findings. Further research is needed to explore the interaction between senescence-related genes and neutrophil infiltration in IDD.

In contrast to previous studies, our research encompasses a comprehensive exploration of mRNA, miRNA, and TFs, enabling a more in-depth understanding of the molecular mechanisms underlying IDD. This approach allows for an extensive examination of gene features and potential regulatory networks related to IDD, providing a unique contribution in comparison to studies that solely concentrate on mRNA. Furthermore, we employed PCR, *in situ* hybridization, and immunohistochemistry to experimentally validate diagnostic markers at both the transcriptional and protein levels. This rigorous approach enhances result reliability and provides valuable confirmation for our findings. Finally, single-cell sequencing analysis found that neutrophils had a very high senescence score, indicating that senescence-related genes may play a role in neutrophils in IDD, further deepening the mechanism research. This methodological difference allows for a more comprehensive examination of gene features and potential regulatory networks associated with IDD, offering new insights into the molecular mechanisms underlying the disease.

Through experimental verification and analysis, we observed discrepancies in the expression trends of certain genes compared to the preliminary analysis. These inconsistencies may be attributed to technical limitations, sample heterogeneity, disease complexity, and small sample size. Technical limitations arise from inherent errors and variability associated with qPCR, which can lead to different expression trends. Sample heterogeneity, including variations in age, gender, disease severity, and treatment status, can influence gene expression and contribute to divergent trends. The complexity of intervertebral disc degeneration (IDD) involves multiple regulatory mechanisms, resulting in varying gene expression among patients. Additionally, the limited sample size limits the accuracy and reliability of our findings, leading to unstable and inconsistent results.

There are some limitations in this study. First, although we have conducted validation using nucleus pulposus tissue, further biological experiments are needed to confirm the clinical value of this study due to the small sample size. In addition, the sample size of this study was limited due to the lack of high-quality datasets, and the analysis results may be biased. Therefore, in future studies, we will continue to expand our sample size and conduct basic experiments to validate our results for better clinical application.

In summary, this study has developed a diagnostic model that significantly associates immune cell infiltration with IDD based on key senescence-related DEGs. Early screening and effective prevention of high-risk populations from the perspective of senescence-related genes will have a profound impact on the management of IDD.

## Data Availability

The original contributions presented in the study are included in the article/Supplementary Material, further inquiries can be directed to the corresponding author.
