# Supplementary material for: Comprehensive analysis of senescence-related genes and immune infiltration in intervertebral disc degeneration: a meta-data approach utilizing bulk and single-cell RNA sequencing data
Source: Front Mol Biosci. 2023 Dec 22;10:1296782. doi: 10.3389/fmolb.2023.1296782 (PMC10770860; doi:10.3389/fmolb.2023.1296782)
Supplement: Supplementary file 3 [file Table1.DOCX]

Supplementary Figure 1


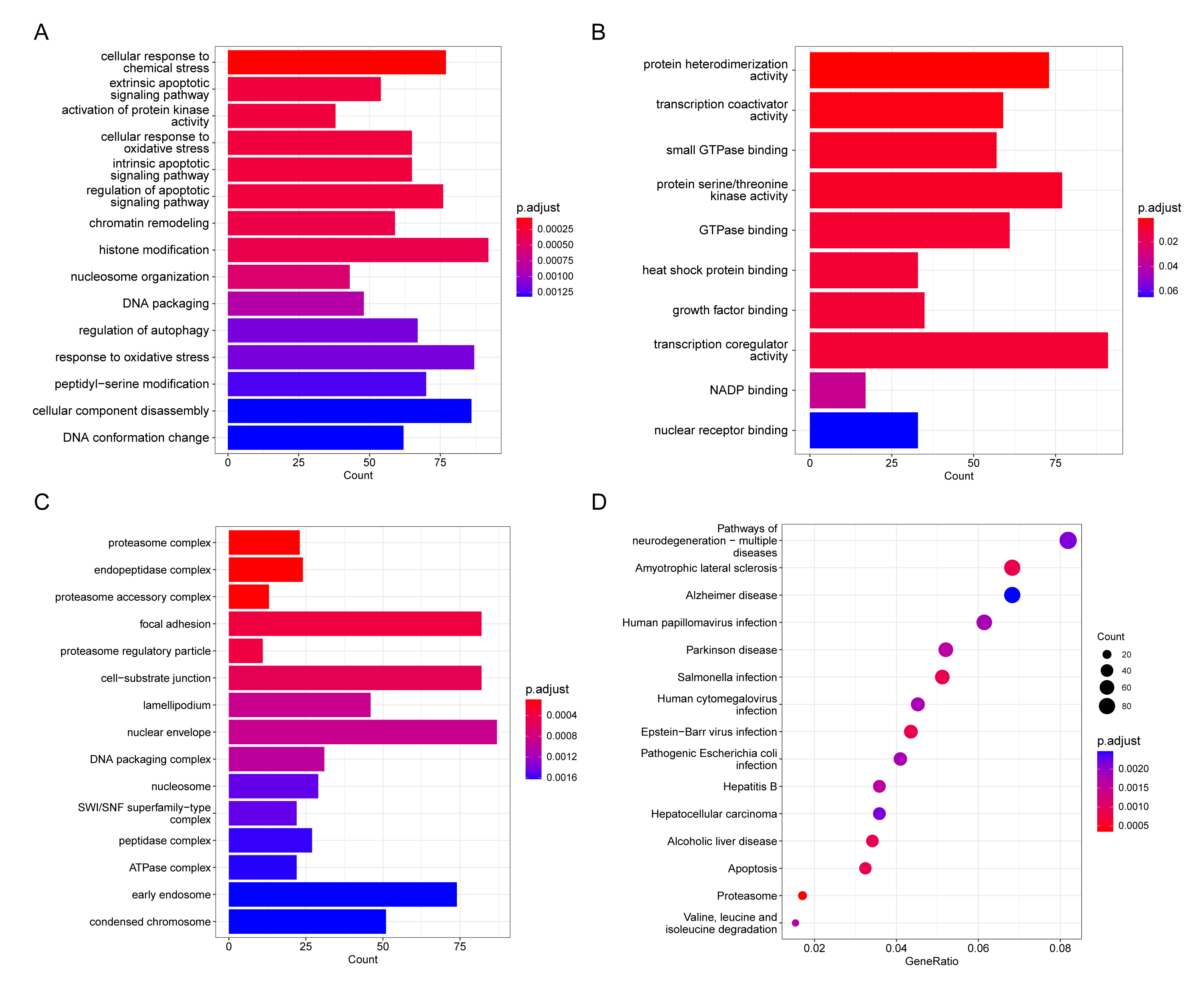


Supplementary Figure 1 Gene Ontology (GO) and Kyoto Encyclopedia of Genes and Genomes (KEGG) enrichment analysis. (A) Biological process (BP), (B) molecular function (MF), (C) cellular component (CC), horizontal coordinate is the number of genes, vertical coordinate is the GO term, and color indicates P.adj. (D) KEGG pathway enrichment analysis, the horizontal coordinate is the gene ratio, the vertical coordinate is the pathway name, the size of the circle indicates the number of genes enriched in the pathway, the color of the circle indicates the P.adj.
